# Sexual violence, secrets, and work: Ruling relations of campus sexual violence policy

**DOI:** 10.1111/cars.12491

**Published:** 2025-01-07

**Authors:** Lindsay Ostridge

**Affiliations:** ^1^ Institute of Feminist and Gender Studies University of Ottawa Ottawa Canada

## Abstract

Campus sexual violence complaints involving students might seem easy to record and report, but university campuses in North America have a culture of secrecy and tend to focus on neoliberal approaches. In this paper, I trace the genealogy of a sexual violence policy from an unnamed university to argue that ruling relations make the current provincially mandated stand‐alone sexual violence policies into a performative tool that silences expert knowledges, coordinates institutional practices towards a particular type of sexual violence prevention, and re‐inforces a broader neoliberal logic in higher education. I explore my argument in the following three sections: the social organization of the policy and prevention campaign, the rules and regulations of the policy, and the neoliberalism of the current sexual violence discourse. As my analytical framework, I draw on Dorothy Smith's social ontology, which aims to investigate the practices and experiences of people by focusing on work and bodily existence as key points of reference. Drawing upon in‐depth semi‐structured interviews I conducted with fourteen participants (and one email exchange) at an unnamed Ontario university, I analyze how variously positioned people within an institutional structure negotiate relations of ruling in the specific context of campus sexual violence.

## French Abstract

Malgré l'éventail des politiques en place, la violence sexuelle sur les campus universitaires nord‐américains demeure un problème aux effets durables. Cet article retrace la généalogie d'une politique de lutte contre la violence sexuelle dans une université en mettant l'accent sur l'organisation sociale de la politique et de la campagne de prévention, sur les règles et règlements de la politique et sur le néolibéralisme du discours actuel sur la violence sexuelle. Les résultats de 14 entretiens semi‐structurés approfondis avec des avec des professeurs, du personnel et des étudiants d'une université de l'Ontario montrent comment des personnes occupant des positions diverses au sein d'une structure institutionnelle négocient des relations de pouvoir dans le contexte spécifique de la violence sexuelle sur le campus. Je montre que les relations de pouvoir font des politiques autonomes de lutte contre la violence s exuelle imposées par la province un outil performatif qui réduit au silence les connaissances des experts, coordonne les pratiques institutionnelles vers un type particulier de prévention de la violence sexuelle et renforce une logique néolibérale plus large dans l'enseignement supérieur.

## INTRODUCTION

Sexual violence on North American university campuses continues to be a problem with lasting effects. Survivors can experience significant trauma and fear of retaliation while being confronted with needing to think about avoiding the perpetrator in multiple residence spaces, classes, and on campus. Also, survivors need to consider potential material losses after the trauma, such as loss of tuition, loss of professional development (teaching and research assistantships), and the cost of healing that might come from therapy or counseling (Sheehy, 2017). Across Canadian universities, students regularly protest the current systems in place. For example, in 2021, Western University students used social media, especially TikTok videos, to disclose sexual violence during frosh week. Thousands of students gathered and walked out of classes to protest their university's lack of action, revealing dissatisfaction with the administrative response (CBC News, 2021).

A recent survey of Ontario university students revealed that approximately 60% of surveyed students did not understand or know of their university's support, services, accommodations, and reporting procedures for sexual violence (Ministry of Colleges and Universities, 2019). Some of the supports of which they were unaware included counseling services, health services, and housing services, as well as academic accommodations. This lack of knowledge is particularly alarming because, in Ontario, all universities are provincially mandated to have a stand‐alone sexual violence policy, training, and prevention program. Universities also need to provide sexual violence prevention materials that are publicly available from the first week of orientation and throughout students’ time at the university. They need to publicly record the number of times students have inquired about reporting sexual violence and to run programs that help ensure safe campuses (Government of Ontario, 2015). So, why, when it appears that so much is available to prevent sexual violence, are students still protesting and demanding change?

Although campus sexual violence complaints involving students might seem easy to record and report, university campuses in North America and beyond have a culture of secrecy (Phipps, 2020; Yung, 2015). Instead, universities tend to focus on neoliberal tactics that rely on self‐securitization, increased security, and individualism while adapting market‐ready logic to the university system (Phipps, 2017; Quinlan, 2017b; Trusolino, 2017). In this paper, I trace the genealogy of a sexual violence policy from an unnamed university to argue that ruling relations make the current provincially mandated stand‐alone sexual violence policies into a performative tool that silences expert knowledges, coordinates institutional practices towards a particular type of sexual violence prevention, and re‐inforces a broader neoliberal logic in higher education.

I explore my argument in the following three sections: the social organization of the policy and prevention campaign, the rules and regulations of the policy, and the neoliberalism of the current sexual violence discourse. As my analytical framework, I draw on Smith's (2005, 2006; Smith & Griffith, 2022) social ontology, which aims to investigate the practices and experiences of people by focusing on work and bodily existence as key points of reference. Drawing upon in‐depth semi‐structured interviews with 14 participants (and one email exchange) with faculty, staff, and students at an unnamed Ontario university, I analyze how variously positioned people within an institutional structure negotiate relations of ruling in the specific context of campus sexual violence. I examine what is supposed to happen in terms of sexual violence prevention and response alongside the everyday experiences of people working hard to prevent and respond to sexual violence in the institutional setting of the university. Therefore, my focus is on “work,” directing my attention to the work processes of conducting sexual violence prevention and response.

## INSTITUTIONAL ETHNOGRAPHY

Canadian Sociologist Dorothy Smith developed institutional ethnography during the 1970s as a feminist research approach (Balcom et al., 2021; Smith & Griffith, 2022), which aimed to work towards a more equitable society by pointing out structural inequalities (Campbell & Gregor, 2002). Branching out from traditional methods of sociology, Smith wanted to hear and validate women's knowledge and experiences, which she found were often subordinated by the authoritative knowledge of male academics and experts. In this process, Smith developed a sociology where anyone, regardless of gender, could seek knowledge instead of relying on authoritative expertise to explain their experiences (Balcom et al., 2021; Campbell & Gregor, 2002). She used people's everyday experiences to learn about the institutions that organize and rule their lives (Devault, 2006). Representing a combination of Feminism and Marxism, institutional ethnography is “an alternate sociology” (Smith, 2005, p. 1).

Many prominent sociologists have since taken up Smith's framework. For example, G. W. Smith (1988) explores how the police control gay men's sexuality, while Rankin and Campbell (2006) investigate ruling relations in nursing, and de Montigny (1995, 2014) examines ruling relations in child protection services within social work. Some feminist researchers have also used this method to investigate campus sexual violence policies (see Gardiner et al., 2021; MacAndrew, 2017). Institutional ethnography, thus, offers a fruitful approach to analyzing power relations and making meaningful change for students on Canadian campuses.

A central tenet of Smith's framework is to understand how work processes are coordinated or mediated by texts and various discourses (Devault, 2006). In their latest book, *Simply Institutional Ethnography*, Smith and Griffith (2022) refer to the textually mediated process as a recipe involving three points—discourse, work, and text—to discover ruling relations. Ruling relations control work processes, and work activities ground social life and organization (Smith & Griffith, 2022). Through the discourse of task force members, action team members and those who administer sexual violence prevention and response, I explore how specific texts of the campus sexual violence campaign coordinate individuals toward a particular type of sexual violence prevention.

Smith and Griffith (2022) advise starting any analysis of work with the body, as work is an embodied experience. Therefore, I focus on how participants speak about their work as an embodied experience to learn about their daily experiences and knowledge (Campbell & Gregor, 2002; Tummons, 2017) and to make visible the invisible social relations that rule them daily. In our conversations, the participants “activate” texts when speaking about their work processes, as texts “create this essential connection between the local of our (and others’) bodily being and the trans‐local organization of the ruling relations” (Smith, 2006, p. 118–119). In conjunction with a critical feminist methodology, I understand that each interview, as an embodied experience, provides a different perspective on the institution, and institutional ethnography helps me to map out these different perspectives to provide an integrated viewpoint of the university (Smith, 1988). One of the principles that I rely on is that “truth can be multiply defined or understood, and that knowledge is political” (Naples & Gurr, 2014, p. 15).

I conducted 14 in‐depth, semi‐structured interviews (and one email exchange) with faculty, staff, and students at an unnamed Ontario university. I asked broad interview questions to help facilitate conversations centered on their experiences but more specifically focused on how they use, engage, and understand the sexual violence policy and related texts. Due to COVID lockdown restrictions, I used ZOOM to conduct interviews. Using Turner's (2006) method, I mapped the social organization of the sexual violence campaign (see Appendix 1 and 2). In what follows, I draw upon shortened transcribed excerpts of participants’ interviews for brevity and clarity, but much of the discourse remains. To better understand an integrated view of the relations of ruling, I also tried to include the words of most of the participants while still selecting those who best revealed the different aspects of the university. All participants’ names are anonymized (for a list of names with demographics please see Appendix 3).

## SEXUAL VIOLENCE IN UNIVERSITIES

Campus sexual violence is an enduring problem affecting universities across the Anglophone North (Phipps, 2024). In the United States, research suggests that 20%–25% of female students experience either a completed or attempted sexual assault (Krebs et al., 2009). A national study of sexual violence in UK universities reveals that one in seven women experiences physical or sexual assault while being a student (Phipps, 2020). The prevalence rates in Canadian universities suggest that the numbers have remained relatively stable across two decades, with approximately 33% of female university students experiencing sexual violence (DeKeseredy et al., 1993; Newton‐Taylor et al., 1998; Quinlan et al., 2017a). More broadly, a recent nationwide 2019 survey revealed that approximately 71% of students witnessed or experienced campus sexual violence (Burczycka, 2020).

Universities have a civic duty to ensure the safety of their students (Towl, 2016); thus, student activists continue to put pressure on universities in North America, the United Kingdom, and Australia to respond and create meaningful change (Colpitts, 2022; Sheehy & Ostridge, 2022). Student perspectives are increasingly important, as demonstrated in films such as “The Hunting Ground,” which features American student survivors speaking about their failed attempts to gain support from their universities (Phipps, 2024; Towl, 2016). A point of contention is that most student activists come to sexual violence with an intersectional feminist approach to harm (Phipps, 2024; Méndez, 2020). However, much of the campus sexual violence scholarly literature, research, and policy is underpinned by a top‐down crime prevention approach, often including police and security responses (Brockbank, 2021; Patel & Roesch, 2018; Phipps, 2024; Quinlan, 2017b).

Correspondingly, there has been an increased focus both locally and internationally on creating policies and legislation to respond to campus sexual violence (Patel & Roesch, 2018; Quinlan, 2017b; Sheehy & Ostridge, 2022). In 2013, the United States Congress passed the Campus Sexual Violence Elimination Act (to work in conjunction with Title IX and the Clery Act) to ensure that all incidents of sexual violence are recorded and made public. Every university is expected to have a Title IX coordinator who records each incident, and some universities expect all staff to engage in mandatory reporting practices. Similar legislative changes have happened at Canada's provincial level, such as mandating universities to create sexual violence policies. Ontario, British Colombia, and Manitoba have made legislative changes; while Alberta and Nova Scotia have yet to do so, they have guided universities to create these policies (Patel & Roesch, 2018). Campus sexual violence policies are supposed to provide an alternative to the criminal justice system, which has problems such as underreporting, failure to investigate, and discriminatory processes (Sheehy, 2017). 

Yet, problematically, there has been a rise of neoliberal ideology and discourse within universities, which works against touchstones of the academy such as civic discourse (Giroux, 2002; Ramírez & Hyslop‐Margison, 2015). Giroux (2002) argues that there is a movement towards the “language of commercialization, privatization, and deregulation and that, within the language and images of corporate culture, citizenship is portrayed as an utterly privatized affair that produces self‐interested individuals” (p. 425). Academics observe how neoliberal ideology and discourse suppress structural dissent by ignoring social injustices and by creating anxious faculty, staff, and students with changes to university funding, employment conditions, and academic freedom (Giroux, 2002; King, 2015; Quinlan, 2017b). For example, the corporatizing of universities allows management to hire off the tenure track, offer fixed‐term contracts, and pay some academic staff hourly, giving rise to a precarious workforce (Phipps, 2024; University & College Union, 2023; Whitley, 2020). In turn, Phipps (2020) suggests that many faculty feel pressure to de‐escalate any complaints of sexual violence in favor of maintaining a positive corporate image and “brush[ing] the issue under the carpet” (p. 230). After the de‐escalation, she argues that the complainant may be discouraged from pursuing allegations, or the perpetrator may be quietly asked to leave, as a single person is “airbrushed” out of the school (Phipps, 2020). Few papers have traced the genealogy of one campus sexual violence campaign from before the legislative changes to after; thus, this research offers valuable insights into the everyday and embodied experiences of variously positioned actors and details how ruling relations organize their lives, inhibiting the meaningful change that students call for.

In what follows, I discuss the case study followed by my analysis in the following three sections: the social relations of campus sexual violence prevention, the rules and regulations of the sexual violence policy, and the neoliberalism of sexual violence.

## CASE STUDY

In 2013–2014, the Canadian media published a large number of articles on sexually harmful actions at universities, which included rape chants, toxic Facebook posts, and complaints of sexual assaults (for example, see CBC News, 2013, 2014; CTV News, 2013). In this coverage, the media raised issues such as accurate prevalence rates of sexual violence and the experiences of women trying to get accommodations through their universities after victimization (Choise, 2014; Ormiston, 2014; Sheehy, 2017; Stermac et al., 2017). As Quinlan et al. (2017a) argue, these stories “draw particular attention to the perniciousness of a rape culture on campus and the inadequacy of Canadian universities’ prevention and response to sexual violence” (p. 1). More specifically, Mathieu and Poisson's (2014) investigative journalism revealed that few universities had a stand‐alone sexual assault policy. Instead, many universities relied on older sexual harassment policies, or student codes of conduct to address sexual assault.

In 2015, Ontario's Liberal government released *“It's Never Okay: An Action Plan to Stop Sexual Violence and Harassment*,” a document it hoped would highlight the problem of sexual violence and harassment in provincial workplaces, including universities. The action plan was supposed to address public outrage over several mishandled sexual violence cases at post‐secondary institutions. Shortly after the action plan was released, the province passed (*Bill 132*, 2016; Government of Ontario, 2016), which required all Ontario post‐secondary institutions to create stand‐alone sexual violence policies by January 1, 2017. The provincial government briefly outlined what universities must include in a stand‐alone sexual violence policy in *Bill 132* (2016). In 2022, the Ontario government made additional changes to the legislation, requiring that all universities not discipline students for alcohol or drug use in acts that involve sexual violence (Ontario Regulation 646/21, 2021).

However, before *Bill 132* (2016) became official, one university came under media scrutiny due to significant incidents of sexual violence involving students. Shortly after news broke of allegations of sexual assault, the university quickly responded: a media conference with a university official and an announcement that the university administration was assembling a task force. This institution started implementing some of the task force recommendations before and during the legislative changes, adding a level of complexity that other Canadian universities might not have experienced. It seemed to be on track to creating a stand‐alone sexual violence policy before the province required it, with subject experts available to implement a culture change in the university environment.

Like other Canadian universities, this university has a history of campus sexual violence. This is made clear by the history described above where, nearly a decade earlier, students called for the university administration to implement procedures to protect students from sexual harassment. First, there was a letter campaign where many challenged the university administration to confront professors who sexually harassed students. In response, a sexual harassment working group was compiled and made recommendations to improve the university community and protect students. Finally, as a result, the university created a student support office and put additional measures in place, such as a sexual harassment policy. I include this history to demonstrate that the university had already experienced previous problems of sexual violence and made moves that seemed quite similar to the more recent response described above.

### The social relations of campus sexual violence prevention

Smith and Griffith (2022) contend that institutional ethnography involves a researcher listening to what people do in their everyday work and how that interacts with others’ work, whether in the past, present, or future. The authors take from Marx and Engels's (1973, 1976) work on investigating the everyday, as it reveals the “relations that overpower their lives” (Smith & Griffiths, 2022, p. 50). Ruling relations, thus, “impose an objectified mode on to us” (Smith & Griffiths, 2022, p. 7). In this section, I focus on the university's response to sexual violence in the media, where the university's administration or management created the task force and action teams tasked with making recommendations and writing the policy. I trace how the institution draws people into particular social relations with each other. I investigate the working connections of sexual violence prevention experts, advocates, and university administration to analyze how they discuss and navigate the ruling relations. I also investigate the notion of “work” as participants discuss what work is valued and not valued by the university administration. Specifically, I look at the university's emphasis on the valued work of legal experts that aligns with the provincially mandated *Bill 132* (2016), and a particular form of sexual violence response.

After a large‐scale media blitz documenting sexual violence at this university, the President publicly announced the university's next steps, one of which included the creation of a task force. Next, as noted in the interviews, the President's office requested individual subject matter experts (faculty and staff) to be on the task force. However, each participant had a different entry point into the group. The experts who worked within the university were privately invited by leadership (e.g., the President or a Dean). Some experts recommended others who worked outside the university community to the President's Office, based on their perceived reputation, expertise, and connection to activism. In terms of student involvement, some student activists nominated themselves due to their previous relationship with the President's Office and some were expected to join due to their roles within the university (e.g., student union representatives).

Many of the members mentioned in interviews that they had mixed feelings about being on the task force. For example, since being on the task force was a serious commitment and hard work that was beyond a service duty, many wanted to be paid for their time. Due to this fact, many members felt that giving their time to this cause was important but were resentful that the university did not compensate them for this task. Some referred to it as a “duty,” an “obligation,” a time to “step up” and show their support for “feminist” leadership. Some were motivated by being in a room with “powerful” people who worked in the area, but they also felt that universities are well‐funded and should put money towards these efforts. The discourse of neoliberalism was very present as the task force members discussed an increase of labour, on top of their current workload since teaching and administrative relief are not generally offered when working on such projects. But for some, it was entirely volunteer work. For example, Samantha, a White cisgendered lesbian student union representative, spoke to the university's expectations and compensation for her labour, saying:

I think it is a lot to ask of people. I think that the only, and I am using air quotes here, “*remuneration”* that we received for being on the committee was a cheese board that I still have. Literally a [university name] branded cheese board, and a nice little reception at the end of it. There was no compensation for time. I was happy to give the time because I was in a position to do so, and I felt it was important, but it is a big role to ask somebody to take on a volunteer basis.

Samantha voices palpable disappointment that this work should be “volunteer” and ridicules this expectation. The suggestion is that the university administration, through the President's Office, sets the parameters of the committee to be exploitative where, ultimately, some members (students) are given a university “branded” honorarium with some social recognition. The task force final report notes that meetings took place over 9 months, and the tasks were considerable. They included writing the final report, conducting an online survey for students, attending open consultations with deans and various services, including sports, health, housing, student rights, Equity Rights Center (ERC), as well as campus unions, and violence against women community‐based organizations. Also included among the tasks were getting expertise and guidance from other Canadian campus sexual violence working groups, as well as commissioning a report from “an independent campus sexual violence social justice advocate.” Finally, task force members were expected to search and review academic literature, both general and specific to this particular university context, to find best practices.

In addition to the expectation of volunteered labour for some, the university administration gave people separate roles within the task force. Sophia, a White, cisgendered heterosexual female professor explains the feeling of not being a “representative group” and how that created a certain dynamic:

We had many meetings, many discussions, and not a lot of disagreement. You know, the thing that was most difficult was the fact that we were not a representative group. There was one racialized woman on the committee and one Indigenous woman who was more an observer than an actual appointee. So, we weren't representative in that sense.

In this specific context, the words “representative in that sense” seem to be about race and equity. Ahmed (2007) thinks of whiteness as an orientation: “If we start with the point of orientations, we find that orientations are about starting points… such a world is the world that we are in, where things take place around me, and are placed around me” (p.150–151). If most of the committee is White, the standpoint is White or, at least, the orientation is White. For the university administration to create an “observer” role for the sole Indigenous person on the committee and only a few appointee roles for racialized people resurrects a settler colonial hierarchy. In addition, there was little to no mention in the interviews of two‐spirit, trans or gender‐diverse members being part of the group.

Along with managing who was part of the groups (task force and action teams), and the roles they had, the administration controlled the discourse/framing of campus sexual violence. For example, Anna, a student union representative, anti‐racism activist and a woman of color, remarks that many of the suggestions put forward by herself and other women of color in the action group (tasked with taking up the recommendations of the task force and writing the policy) were not taken up. Because of this, she recommends *more* gatekeeping, but specifically of the President's office:

I just felt like there needed to be some kind of gatekeeping on this conversation. There were certain things that the President's university administration probably did not agree with, and they wanted to ensure that there were like‐minded individuals who would make sure that certain things would not be addressed or brought into the policy and protocols for the university. And so, they had to basically give power to someone whom they understood would think and make decisions just like them and who probably was consulting them on every decision, piece, and item before coming to the table, which was later revealed that that is what was happening.

Anna comments on ruling relations within the group's everyday conversations. She calls attention to the significant (and secretive) role of the President's office in selecting like‐minded people who would restrict discussions to align with the President's office. Anna's representational presence is all that the university requires, without any need to actually listen to her concerns. She is an unpaid student union representative expected to attend these meetings as part of her role. The neoliberal logic of time efficiency restricts discussions and quickens timelines while excluding the possibility of having deeper conversations that might involve intersectionality (King, 2015). Student input does not need to be heeded, but even so fulfills the requirements of *Bill 132* (2016), which states that a college or university “shall ensure that student input is considered, in accordance with any regulations, in the development of its sexual violence policy and every time the policy is reviewed or amended” (Bill 132, 2016, Student Input, Section 4). Anna further describes dropping out of the process by not attending meetings. She said she felt cautious and singled out as an “angry black woman” when working with campus administration.

Overall, many task force and action team members expressed disappointment in interviews with the lack of participation from students. Confused about why this was happening, some thought it was a lack of interest, trust, or a feeling of powerlessness within the institution. But perhaps it was that students disagreed with the particular brand of feminism that was being employed. Charlotte, a White, heterosexual cisgendered legal professor tasked with writing the policy notes:

I think students did not trust the university to make a difference. There was a concern that protection services was not a feminist response to sexual violence. Complainants were not going to call protection services and go through a complaint process that was run entirely by the university. The university was responsible for creating a culture where sexual violence happened, and therefore, it could not also be responsible for fixing that culture.

Charlotte speaks to ruling relations working through the administration and professors’ choices to not support students’ experiences, such as setting up a policy to work with protection services and a complaint process that the university oversees and controls (see Appendix 1 and 2). The university has established a policy that protection services must be contacted in an emergency. In a nonemergency event, the ERC must be contacted. As well, protection services must contact the ERC after they have been reached. All in all, the ERC must be contacted if sexual violence occurs on campus, which reports to the campus administration. Therefore, the administration's ability to entirely control the process can enable the university to engage in “airbrushing” to secure a positive university image (Phipps, 2020, p. 230).

Nevertheless, some voices were actually heard in the consultation process. For example, as a paid social justice advocate (and a White cis‐gendered heterosexual woman), Caroline's expertise fell in line with the campus administration's directive:

My voice was taken seriously, even though it was myself and basically a few student leaders but largely legal folks. My voice as a non‐lawyer, as a non {university name} student was included to provide that survivor perspective. The legal team was looking at it from a liability lens, “We have to protect ourselves”. I was there to say, “But that process that you are proposing is really harmful to survivors, so can we meet halfway and have something that absolutely respects the rights of the accused but also does not abandon the survivor who is making the complaint”.

While Caroline speaks about survivors, she also speaks about the university's rationale to approach the policy from a legal, “protective” framework. Because she is dominated by this discourse, in order to be heard she needs to meet the administration halfway, even though what is being proposed is, in her view, “really harmful to survivors.”

The social organization of the campaign strongly suggests that the university administration, as institutional actors, guided the entire process by selecting certain task force and action group members, and then by listening to some during the consultation process, and only partially to others.

### Sexual violence: The rules and regulations of the policy

The aim of the sexual violence policy was to provide students with a documented pathway to disclosing campus sexual violence and to declare a university commitment to “zero tolerance” of sexual violence. But this document provides an institutionally organized course of action for students and prevention workers. It falls in line with the requirements of *Bill 132* (2016) as it “sets out the process for how the college or university will respond to and address incidents and complaints of sexual violence involving students enrolled at the college or university and includes the elements specified in the regulations relating to the process” (Section 17, 3b). Although the written document fulfills these requirements, it does not include the intersectional feminist thinking present in the committee meetings. Instead, liberal feminism is taken up and student's needs are overlooked as the social structures of race, class, and gender are not considered. Therefore, the document has gaps and oversights that significantly marginalize some students, even if unintentionally. In this section, I discuss how participants’ work of responding to students was, first, coordinated by a policy that sees some and ignores others; and second, was intended to get students and staff to (mandatory) report all cases of sexual violence to the ERC office, which reports to the president's office.

Devault (2006) reveals that management texts provide rules and regulations to be followed, controling the work processes of people providing a service. In this case, the policy acts as a management text that provides rules and regulations that the ERC officers and others must follow. However, following the rules and regulations of the policy is difficult and limiting. For example, Arathy (a cis‐gendered woman of color) speaks to her experiences of working at the Equity Rights Office and the problem of a legal or liability lens:

To be able to take [the policy] and do it, but at the same time say that we are a survivor‐centric involves a lot of compromise, a lot of work around, a lot of rethinking. What does that mean, “we follow the law, and we follow what is written in the *Ontario Human Rights Code*, but we are also survivor‐centric?” There are limits to that. It is not blatant. Being able to have that conversation, learn what that means, and at the very same time communicate that to people who are going through trauma is complicated and requires a lot of mental work.

Arathy demonstrates how her work is textually mediated by the stand‐alone sexual violence policy, *Bill 132* (2016) and the *Ontario Human Rights Code*. These texts control her interactions with students and institutionalize their sexual violence disclosures. Being “survivor‐centric” serves as a placeholder for responding to specific intersectional needs of students that come to her office; but in her view, survivor‐centric approaches and the legal system are opposed. She describes a gap in what we would call the “doing” of the document, and she is left with the mental work of filling this gap by having to explain these limitations to students. Because she is equally tied to survivor and management needs, she attempts to mediate this space as part of her work.

In terms of understanding the role of policy documents, Ahmed (2012) argues it is important to focus on “what they do: how they circulate and move around” (p. 6). The policy discourse can be placed in other forms, such as the university website, where students are able to read, learn, and understand at their own pace what steps and options are available to them. But the policy and related documents can also guide survivors into taking actions that legally protect the university. So, while the policy states that students are encouraged to contact the various offices such as the ombudsperson offices, student's rights, and other student centers such as the women's center and pride center, the website states that the ERC “should” be the only place to go to inquire about sexual violence. For example, Lydia, the ombudswoman (a White, cisgendered female, heterosexual lawyer) said:

In theory, students could contact the ombuds office before or after going to the ERC. In practice (in the three years I have been at {the university}), I have had questions a couple of times from students looking for information about options, support services or accommodation. It is rare that they would come to us for these questions because the information on the university website is quite clear about the ERC being the point of contact. 

Because the website serves as a management text, it coordinates students into believing that the ERC is the “only” place to go, as the website is “quite clear.” It is effective in its dominating power as only a few students have come to the ombudsperson for information about the process of disclosing sexual violence; most ask about accommodations, appeals, and so forth. This is the same as other services at the university, as revealed in interviews. For example, Theodore, a White, cisgendered, heterosexual male student rights advocate (who is trained as a lawyer), notes:

I, personally, have not had a case. I would say it is really rare coming from teachers. This is not to say that it does not exist, but we do not get it often. Usually, if it is really a case of sexual assault, I would maybe tell them to go directly to the legal clinic or something like that.

Again, it appears that few to no students access services to seek guidance around campus sexual violence. Instead, most of the cases that the Student Rights Center gets are requests for “guiding students through various appeal processes such as deferring an exam, asking for retroactive withdrawal due to extenuating circumstances, dealing with allegations of fraud, and mandatory withdrawal” (Peter, heterosexual, cisgendered man of color). In other words, students are following the “institutionally organized course of action” (Smith & Griffith, 2022, p. 94) as the administration, along with others, presents the steps listed in the sexual violence policy as the only course of action.

Moreover, the administration is not giving vital sexual violence prevention training to services that might get disclosures. For example, Ava (a queer, cis‐gendered, woman of color, front‐line response worker at the women's center) notes:

I think they need to hire more experts and representatives for the different departments and different places. They are not very educated. We have had discussions with people coming from [the office of academic support]. I was like, “You should know better than this. You should be educated in this. You should be providing these services”. These are just basic services like your counsellors should be all trained in sexual violence response. Even some of them on the committee do not even have bystander training. I am like, how are you on this committee? You do not have bystander training, and it is the most basic.

Ava speaks to the university's lack of sexual violence prevention training, for example, untrained counselors and unprepared staff. The university is responsible for training faculty on sexual violence prevention and ensuring that training is up to date. Quinlan (2017b) argues that “cost‐efficiency is a guiding principle applied to campus programs, quantified outcomes are compared, and cost‐benefit metrics are used to evaluate potential returns” (p. 63). This means that training could be reserved for the few who work for the ERC because it is cost‐effective to train fewer people, but easier to control the number of disclosures.

Also, it could be beneficial to the university for staff to *not* understand the policy and therefore, to rely on ERC experts to analyze each situation. For example, the university administration makes space for the institution to act on disclosures and pursue investigations without the participation of the survivor. In the case that a “university community member” may be at risk of harm, a “person” is at risk of harm, or an individual is at risk of harming an “identified person,” the university “must” fulfill its legal obligation to investigate the case (University Policy). This caveat suggests that disclosures could be considered reporting once spoken aloud. If this is correct, then a survivor does not control the outcome of the complaint and can be cut out of the process. According to Arathy:

How can I expect a professor in biology, a student in psych, or a counsellor to be able to have read the policy once or twice and be able to say this is what it means? I can't. The policy is not accessible. This is complicated stuff to write a policy on, and it is hard to have a simple, “hey just do this or just do that”, like we can, but I think that is where you have to have resources to go out and explain policy.

Arathy comments on how the policy was written in a way that is difficult to understand and requires “experts” to decipher next steps. There is the option to explain the policy to the community; however, the resources to explain the policy are not offered by the university. This is another way for ruling relations, acting through institutional actors at the university, to control disclosures of sexual violence by making the policy so difficult to understand that students are required to speak to the ERC, who hold the power to act on disclosures without their permission. This institutional course of action leaves little choice for students to disclose sexual violence without having to report it. As a result, few students reach out to this office, and other staff do not understand the policy to provide help and assistance outside of the ERC.

### The neoliberalism of sexual violence

In this section, I discuss the effects of neoliberalism on sexual violence prevention, where the result is that faculty and students experience a culture of fear, precarity, and institutional airbrushing. A neoliberal approach to social problems contains elements of “pluralism, rational choice and institutionalism” (Antony et al., 2017, p. 2). It is a tendency to “see society in individual terms” (Antony et al., 2017, p. 2), with weight placed on the individual choices that people make, because they are expected to make the best choices for themselves. Freedom, autonomy, and choice are paramount to neoliberal thinking as well as the ideology of a just social world. As universities aim to “empower” students by responsibilizing them to make better choices with programs such as the bystander initiative (Colpitts, 2022), they sweep individual cases of sexual violence “under the rug” (Phipps, 2020, p. 230). Phipps (2020) states, “institutional airbrushing reflects particular norms and values, and an attendant reconfiguration of subjectivities…within the neoliberal university environments” (p. 231). There is a:

General fabrication of image over substance, and in a context also characterized by constant monitoring and precarity which have a silencing effect, it is likely that sexual harassment and violence are among many issues we overlook while we try to keep our jobs (at best) or further our careers (at worst) (Phipps, 2020, p. 231).

This fabrication of image over substance affects more than just survivors and perpetrators, as the precarity of the work environment creates a climate of fear, leading some to be more concerned about their own status and job security than about violence (Phipps, 2020). The recognition of one's own precarity can produce a silencing effect on sexual violence prevention and response: from staff outside the ERC not wanting to discuss sexual violence and actively avoiding participation in sexual violence programming, to telling others to protect their careers and not disclose sexual violence (as stated in interviews). Campus administration re‐inforces this precarity by stepping in and making decisions on sexual violence cases secretly. They keep others unaware of how they are handling cases, make official public reports vague, and internal reports secret and difficult to use, all to keep the official numbers of reported cases of sexual violence down. This maintains the neoliberal notion that sexual violence is a problem of individual “bad apples” and not the institution's problem to solve.

The task force and action teams both recommended campus‐wide sexual violence prevention training. This included ERC response workers going into classes and residences to introduce students to the center, as well as creating social activities. Yet, the ERC response workers found it difficult to coordinate with others. Emma (a White, cisgendered heterosexual woman and ERC response worker) discusses the reluctance to have that conversation with other departments:

When I started, I tried to connect with different services, meet with them. But they were not ready to talk about it, or they did not know how. So, it was hard to connect with other services to say, ‘Hey, can we work in collaboration to offer an activity around that?’

Emma speaks about how others appear uncomfortable around conversations about campus sexual violence. Her first year of enacting the prevention program was the most precarious because the context of the campus environment meant that people did not want to discuss sexual violence. Just previously, the cases of sexual violence were in the press nationally. All university staff and students would be aware of the task force, action teams, and the creation of the stand‐alone sexual violence policy. So, it seems strange to Emma that people did not want to talk about it.

As students, staff, and faculty are seemingly aware of the institutional course of action, there is an expectation by an administration that sexual violence prevention and reporting is part of your job, although it is not a wanted part of the job. Arathy explains this:

There's a little piece in our policy that says the expectation of everyone who works for the university, if a disclosure is made, should refer the person to the ERC. But they do not know whether they should be sending people. So, a lot of times, out of miseducation, they will say things to survivors that you cannot backtrack from. They give their own personal advice, or they talk about how they dealt with it and how it did not really work. So, instead of allowing a survivor to choose how they want to proceed, the idea of ‘hush it up, do not talk about it, there is actually nothing that can be done’, and ‘protect your career’ gets reinforced.

Arathy speaks to the actual feelings of some members of the campus community gained through personal experiences, and their advice of “hush it up, do not talk about it, there is nothing that can be done.” Again, the policy states mandatory referral reporting to ERC, and if people do not do that, they might risk their career. Ahmed (2015) writes about sexual harassment as an institutional problem:

If we speak of sexual harassment as organizational culture, we threaten the organization's reputation. Those who are damaged become the ones who cause damage. And the institutional response can take the form of damage limitation. There are so many ways those who speak about harassment, whether their own harassment or the harassment of others, are silenced: you don't even need to sign a confidentiality agreement to be warned of the consequence of your actions. And then, too often, the ones who are harassed end up being removed or removing themselves: if the choices are “get used to it” or “get out of it”, some quite understandably “get out of it” (para 3).

In taking up Ahmed's (2015) notion that speaking about sexual harassment or sexual violence causes damage, professors and others at the university might very well be not speaking to “get out of it.” While fully being aware of their duty to inform the complainant of the ERC and what is expected of them by the university administration, which is to hand over information to the administration, they do not do so, perhaps for fear of reprisals.

Even for those who do speak out about sexual violence and work within the university framework as a review committee member, there is the same sentiment from participants of wanting to “get out of it” when faced with “get used to it.” For example, Daniel (a White, queer cisgendered male student volunteering on the sexual violence review committee) comments on the feeling of institutional silencing when working on a case as a sexual violence review committee member:

I have to say, not knowing what happens after your process is done, you hand in your report back to the ERC, in theory, they just send it right away to the dean. You have no idea what happens, but that leaves you a bit in the dark, like, “Oh, I wonder what happened”?

Daniel comments on the institutional course of action that gives university deans (or the appropriate authority) the power to decide the outcome, and he is left wondering if his work makes an impact. The current institutional framework makes the process entirely internal, with the performative aspect of including a sexual violence review committee. He later mentions his frustrations with one dean's power to override decisions made by the sexual violence review committee:

I think that is the wrong way of approaching it, that you can just override and say, “Meh”. In the last year or two, the ERC has been more involved in trying to limit some of the things that we see, which I thought was unfortunate. I did my last review committee in the summer, and I do not think I will do another one because of that experience.

Daniel chooses not to continue to participate because he is being asked to “get used to it” while working on files that are heavily controlled by the ERC. It is important to note that the ERC reports to the university president's office. The ERC acts as management by further limiting information within the university community. Daniel later comments on seeing a professor accused of severe sexual harassment of a student, walking around on campus months after his case was reviewed, suggesting that the committee's recommendations were disregarded. Phipps (2020) argues that when complaints are made against professors with high grant income or who are “star” professors, the sexual violence complaint is not taken seriously by the university. Instead, the university gives power and assigns more value to those who provide economic resources to the institution, protecting them.

Additionally, many of the people who are involved in the creation and implementation of the sexual violence policy and program are left out of knowing the results of their work or how their work is being used once the administration takes over. Most participants spoke about wanting to know what is happening because the reports from the university never seem to give a clear picture. During an interview, Sophia (a White, cisgender, heterosexual, woman) states the following: “I don't know how it's panned out. I don't know how it has been implemented, and that is always an anxiety. You put a lot of work into a policy, and I have no idea how it is being implemented.” Another participant, Anthony (White, heterosexual, cisgender male, senior administrator) states the following:

I could not tell you how many complaints there have been of sexual violence or what the outcomes have been. I am not really in touch with students on the level where I can say, “How are you feeling about things in this regard?”

This fragmentation of work processes comes across as secretive and underhanded by campus administration, to the frustration of the many participants who want to see full and honest reports of students' experiences of launching complaints. For example, Sophia comments the following:

I would like to see a full report on what is happening. How many students have launched complaints? What has happened to those complaints? How do the students feel about it? How are they resolved? How were they investigated? How are they treated both in terms of responding to the allegation and in terms of the support and accommodation that they requested? I have seen reports from other universities where students are really still incredibly unhappy and feel abused by the process.

Sophia asks to see specific metrics on how the policy is being implemented. She is asking for an analysis of what students are experiencing and what they feel about it rather than general statistics. Some of these metrics are being collected but Ava explains how the administration withholds internal working documents:

The data set is huge. It is very specific‐ how far processes go and who signed off on it. But what they give out is just general statistics. The ERC probably does not get a quarter of the actual instances of sexual violence because not all of the services that are supposed to use it, use it. Not a lot of people feel trustworthy inputting there. Also, the process is weird, because sometimes we have to refer people who come to us to ERC, but then if we record it, and they record it, the data doesn't make sense. And, honestly, people come here and say, “I do not want to do anything about this. I just want to talk”. So, we do not record it.

Ava reveals that the university creates one report for the general public and one for the administration. The internal file is a much larger file with specifics regarding each step of a reported sexual violence case. This document serves as a monitoring system to keep numbers down, as it is difficult to use. She comments on the ambiguities of this reporting system as it does not account for the natural movement of students. In this way, Ava speaks to the complicated process of “when” to record data for the university as a front‐line worker.

It is the standardization or institutionalizing of a process that requires nuance and judgment. Charania (2010), in her ethnographic analysis of an all‐girls progressive school, draws on Patricia Williams's (1991) argument that institutions “[make] up its own breed of narrower, simpler but hypnotically powerful rhetorical truths' to avoid complications that emerge when dealing with life and human beings” (as cited by Charania, 2010, p. 322). In a similar vein, the university takes up a process based on a simpler truth of recording sexual violence, which creates a system that makes both students and staff distrust the university.

In sum, the university approaches sexual violence from a neoliberal framework. Participants speak to a culture of fear and precarity resulting in staff wanting to avoid speaking about sexual violence. They are asked by the institution to “get used to it,” or “get out of it” (Ahmed, 2015). The culture produces a silencing effect because people fear speaking about sexual violence. The university, thus, operates in a patriarchal top‐down fashion where the campus administration makes decisions that are economically beneficial to the university, while not offering improvements towards staff and students well‐being and safety (Phipps, 2020).

## CONCLUSION

My purpose was to investigate relations of ruling by tracing the genealogy of one university's stand‐alone sexual violence policy, from the formation of the task force, and subsequent action groups, to the implementation of the policy, to the proposed future changes. I argued that the stand‐alone sexual violence policy that emerged is largely performative. From the start, the social organization of the task force and action team committees included management's expectations of increased labour, institutional whiteness, and performance culture to engage the legal language of *Bill 132* (2016) and supporting a neoliberal approach to campus sexual violence. In terms of the “doing” of the policy and prevention campaign, the gaps, oversights, and marginalization are apparent in the everyday running of the policy and prevention programs. In this space, the first‐line responders struggle with finding ways to communicate the gaps to students between survivor‐centric responses and what is printed in the policy. The policy rules and regulations limit survivors’ options, as the institutional course of action is to go to the ERC, where any disclosure could become a formal report. Because students do not trust the ERC, which reports to higher levels of management, few students seek help or accommodations, and staff do not know how to respond due to the unspoken mandatory reporting system. The culture of a university employing neoliberal logics also means that there is a climate of fear, precarity, and secrecy that enables the university to engage in institutional airbrushing. Ruling relations, working through activated texts, control all aspects of the institutional course of action, which is designed to regulate individuals rather than address the systemic problem of sexual violence.

The stand‐alone sexual violence policy and *Bill 132* (2016) are good examples of how texts can become performative tools used to coordinate the actions of those involved in providing a particular type of campus sexual violence prevention and response, reflecting and reinforcing broader neoliberal logics in higher education. As seen in the discourse of the 14 participants (and one email exchange), these activated texts are used to display “management” narratives aimed at subordinating expert knowledge and creating an administratively difficult process to keep official numbers of sexual violence low. Due to the confidential nature of the topic, the university can “hush up” the official numbers and have its own take on making public metrics on campus sexual violence to maintain a good corporate image, thereby advancing the economic and legal benefits to the institution. Using a critical feminist methodology to challenge notions of universality, I identified some of the processed “underlying oppression and subjugation” in this particular context (Frost & Elicaoff, 2014). My analysis revealed that many stakeholders on campus are oppressed and subjugated by the ruling relations working through the performative nature of this policy, which appears to take up a feminist discourse albeit liberal feminist but is actually rooted in a neoliberal framework.

This is not to suggest that *Bill 132* (2016) and the stand‐alone sexual violence policy are not working at all; they may very well be working for some students, mainly those who fall within a mainstream feminist framework of sexual violence, and those who can make “good personal choices” within the neoliberal approach. But without full disclosure of the processes in terms of reporting how many students ask for information, the university community is either unable to ascertain the extent of the problem fully, or it is permitting the university to engage in erasing the inquiries of both students and professors. What ends up happening is that people go to the media to disclose the wrongdoings of their university and engage in “outrage culture” (Phipps, 2020, p. 235), further individualizing the problem of sexual violence, and creating an ideological circle where sexual violence is erased as a systemic problem. Disclosing sexual violence is meant to engage in accountability for both the university and perpetrators on a systemic level, and finally *stop* the ruling relations from objectifying people's lives (Smith & Griffith, 2022).

## CONFLICT OF INTEREST STATEMENT

The author declares no conflicts of interest.

## Supporting information



Supporting information

Supporting information

## References

[cars12491-bib-0001] Ahmed, S. (2007) A phenomenology of whiteness. Feminist Theory, 8(2), 149–168.

[cars12491-bib-0002] Ahmed, S. (2012) On being included: racism and diversity in institutional life. Durham, NC: Duke University Press.

[cars12491-bib-0003] Ahmed, S. (2015) *Sexual* harassment. Feminist Killjoys, December 3. Available at: https://feministkilljoys.com/2015/12/03/sexual‐harassment. Accessed 5 June 2023.

[cars12491-bib-0004] Antony, W.A. , Samuelson, L. & Antony, J. (2017) Social problems and social power. In: Antony, W. , Samuelson, L. & Anthony, J. (Eds.) Power and resistance: critical thinking about Canadian social issues, 6th edition. Blackpoint, Nova Scotia: Fernwood Publishing, pp. 1–21.

[cars12491-bib-0005] Balcom, S. , Doucet, S. & Dubé, A. (2021) Observation and institutional ethnography: helping us to see better. Qualitative Health Research, 1(8), 1534–1541. Available from: 10.1177/10497323211015966. PMC8278555 34092144

[cars12491-bib-0006] Bill 132 . (2016) An Act to amend various statutes with respect to sexual violence, sexual harassment, domestic violence and related matters, 1st Sess, 41st Leg, Ontario.

[cars12491-bib-0062] Brockbank, M. (2021). “Well‐intentioned in some of the most dangerous ways”: Examining Bill‐132 and the subsequent formation of Ontarian university sexual assault policies. Journal for Social Thought, 5(1), 1–14.

[cars12491-bib-0007] Burczycka, M. (2020) Students' experiences of unwanted sexualized behaviours and sexual assault at post‐secondary schools in the Canadian provinces, 2019 . Statistics Canada. Available at: https://www150.statcan.gc.ca/n1/pub/85‐002‐x/2020001/article/00005‐eng.htm. Accessed 24 June 2023.

[cars12491-bib-0008] Campbell, M.L. & Gregor, F.M. (2002) Mapping social relations: a primer in doing institutional ethnography. Lanham, MD: Rowman and Littlefield.

[cars12491-bib-0009] CBC News . (2013) 6 sex assaults on UBC campus appear connected: RCMP . October 29. Available at: https://www.cbc.ca/news/canada/british‐columbia/6‐sex‐assaults‐on‐ubc‐campus‐appear‐connected‐rcmp‐1.2286882. Accessed 25 November 2023.

[cars12491-bib-0010] CBC News . (2014) Ottawa students speak out about ‘rape culture’ . March 4. Available at: https://www.cbc.ca/news/canada/ottawa/ottawa‐students‐speak‐out‐about‐rape‐culture‐1.2558979. Accessed 25 November 2023.

[cars12491-bib-0011] CBC News . (2021) Thousands of western university students walk out to support survivors of sexual violence . September 17. Available at: https://www.cbc.ca/news/canada/london/western‐students‐walkout‐1.6179573. Accessed 25 March 2023.

[cars12491-bib-0012] Charania, M.M. (2010) Reading the body: the rhetoric of sex, identity and discipline in girls’ education. International Journal of Qualitative Studies in Education, 3(3), 305–330.

[cars12491-bib-0059] Choise, S. (2014). University sexual assault codes of conduct could be unfair, law experts warn. *Globe and Mail* , December 30 Retrieved from http://www.theglobeandmail.com/news/national/university-sexual-assault-codes-of-conduct-could-be-unfair-law-experts-warn/article22234993/ Accessed 24 February 2023.

[cars12491-bib-0014] Colpitts, E.M. (2022) ‘Not even close to enough’: sexual violence, intersectionality, and the neoliberal university. Gender and Education, 4(2), 151–166.

[cars12491-bib-0015] CTV News . (2013) Student president calls sexual assault chant ‘biggest mistake I've made’ . September 5th. Available at: https://www.ctvnews.ca/canada/student‐president‐calls‐sexual‐assault‐chant‐biggest‐mistake‐i‐ve‐made‐1.1441458?cache=. Accessed 25 March 2023.

[cars12491-bib-0016] DeKeseredy, W.S. , Schwartz, M.D. & Tait, K. (1993) Sexual assault and stranger aggression on a Canadian university campus. Sex Roles, 8, 263–277.

[cars12491-bib-0017] Devault, M.L. (2006) Introduction: what is institutional ethnography? Social Problems, 53(3), 294–298. Available from: 10.1525/sp.2006.53.3.294

[cars12491-bib-0060] de Montigny, G.J. (1995). Social working: An ethnography of front‐line practice . Toronto: University of Toronto Press.

[cars12491-bib-0061] de Montigny, G.J. (2014). Doing child protection work. In D.E. Smith and S.M. Turner (Eds.), Incorporating texts into institutional ethnographies, 173–94. Toronto: University of Toronto Press.

[cars12491-bib-0018] Frost, N. & Elichaoff, F. (2014) Feminist postmodernism, poststructuralism, and critical theory. In: Hesse‐Biber, S.N. (Ed.) Feminist research practice. Thousand Oaks: Sage Publishing, pp. 42–72.

[cars12491-bib-0019] Gardiner, R. A. , Chisholm, J. & Finn, H. (2021) Translating gender policies into practice: mapping ruling relations through institutional ethnography. In: Stead, V. , Elliot, C. & Mavin, S. (Eds.) Handbook of research methods on gender and management. Cheltenham: Edward Elgar Publishing, pp. 86–101.

[cars12491-bib-0020] Giroux, H. (2002) Neoliberalism, corporate culture, and the promise of higher education: the university as a democratic public sphere. Harvard Educational Review, 72(4), 425–464.

[cars12491-bib-0021] Government of Ontario . (2015) It's never okay: An action plan to stop sexual violence and harassment . Toronto, ON: Queen's Park Press. Available from: https://docs.ontario.ca/documents/4593/actionplan‐itsneverokay.pdf.

[cars12491-bib-0022] Government of Ontario . (2016) It's never okay: an action plan to stop sexual violence and harassment, progress report 2015–2016 . Toronto, ON: Queen's Park Press. Available from: http://www.ontario.ca/page/its‐never‐okay‐action‐plan‐stop‐sexual‐violence‐and‐harassment‐progress‐report‐2015‐2016.

[cars12491-bib-0023] King, T.L. (2015) Post‐identitarian and post‐intersectional anxiety in the neoliberal corporate university. Feminist Formations, 27(3), 114–138.

[cars12491-bib-0024] Krebs, C.P. , Lindquist, C.H. , Warner, T.D. , Fisher, B.S. & Martin, S.L. (2009) College women's experiences with physically forced, alcohol‐or other drug‐enabled, and drug‐facilitated sexual assault before and since entering college. Journal of American College Health, 7(6), 639–649. 10.3200/JACH.57.6.639-649 19433402

[cars12491-bib-0025] MacAndrew, S. (2017) Mishandled: Turnhill University's approach to sexual violence . Social Justice and Equity Studies, Brock University. MA dissertation.

[cars12491-bib-0026] Marx, K. & Engels, F. (1973) Feuerbach: opposition of the materialist and idealist outlooks. London: Lawrence and Wishart.

[cars12491-bib-0027] Marx, K. & Engels, F. (1976) The German ideology. Moscow: Progress.

[cars12491-bib-0028] Mathieu, E. & Poisson, J. (2014) Canadian post‐secondary institution failing sexual assault victims . Toronto Star, November 20. Available at: https://www.thestar.com/news/canada/canadian‐post‐secondary‐schools‐failing‐sex‐assault‐victims/article_963d6d86‐7781‐54d9‐bb18‐82714131f8ca.html. Accessed 20 February 2023.

[cars12491-bib-0029] Méndez, X. (2020) Beyond Nassar: a transformative justice and decolonial feminist approach to campus sexual assault. Frontiers: a Journal of Women Studies, 41(2), 82–104.

[cars12491-bib-0030] Ministry of Colleges and Universities . (2019) Student voices on sexual violence survey . King's Printer for Ontario. Available at: https://www.ontario.ca/page/student‐voices‐sexual‐violence. Accessed 12 March 2023.

[cars12491-bib-0031] Naples, N.A. & Gurr, B. (2014) Feminist empiricism and standpoint theory: approaches to understanding the social world. In: Hesse‐Biber, S.N. (Ed.) Feminist research practice. Thousand Oaks: Sage Publishing, pp. 14–41.

[cars12491-bib-0032] Newton‐Taylor, B. , DeWitt, D. & Gliksman, L. (1998) Prevalence and factors associated with physical and sexual assault of female university students in Ontario. Health Care for Women International, 19, 155–164. 9526335 10.1080/073993398246485

[cars12491-bib-0033] Ontario Regulation 646/21 . (2021) Sexual violence at colleges and universities . Available at: https://www.ontario.ca/laws/regulation/r21646. Accessed 25 March 2023.

[cars12491-bib-0034] Ormiston, S. (2014) Universities under pressure to combat sexual misconduct on campus . CBC News, September 29th. Available at: https://www.cbc.ca/news/universities‐under‐pressure‐to‐combat‐sexual‐misconduct‐on‐campus‐1.2781455. Accessed 24 February 2023.

[cars12491-bib-0035] Patel, U. & Roesch, R. (2018) Campus sexual assault: examination of policy and research. Journal of Aggression, Conflict and Peace Research, 10(2), 103–111.

[cars12491-bib-0036] Phipps, A. (2017) Speaking up for what's right: politics, markets and violence in higher education. Feminist Theory, 8(3), 357–361.

[cars12491-bib-0037] Phipps, A. (2020) Reckoning up: sexual harassment and violence in the neoliberal university. Gender and Education, 2(2), 227–243.

[cars12491-bib-0038] Phipps, A. (2024) ‘Holding on’ in a crisis: theorising campus sexual violence activism within precarious labour relations. Feminist Theory, OnlineFirst, 1‐20. 14647001241232260.

[cars12491-bib-0039] Quinlan, E. (2017a) Sexual violence in the ivory tower. In: Quinlan, E. Quinlan, A. , Fogel, C. & Taylor, G. (Eds.) Sexual violence at Canadian universities. Waterloo: Wilfred Laurier University Press, pp. 1–23.

[cars12491-bib-0040] Quinlan, E. (2017b) Institutional betrayal and sexual violence in the corporate university. In: Quinlan, E. , Quinlan, A. , Fogel, C. , Taylor, G. (Eds.) Sexual violence at Canadian universities. Waterloo: Wilfred Laurier University Press, pp. 61–75.

[cars12491-bib-0041] Ramírez, A. & Hyslop‐Margison, E. (2015) Neoliberalism, universities and the discourse of crisis. L2 Journal: An Open Access Refereed Journal for World Language Educators, 7(3), 167–183. Available from: 10.5070/L27323492. https://escholarship.org/uc/item/2gx093rz

[cars12491-bib-0042] Rankin, J.M. & Campbell, M.L. (2006) Managing to nurse: inside Canada's healthcare reform. Toronto: University of Toronto Press.

[cars12491-bib-0043] Sheehy, E.A. (2017) Making universities safe for women: sexual assault on campus. Previously published as a chapter in In: Antony, W. , Antony, J. & Samuelson, L. (Eds.) Power and resistance: critical thinking about Canadian social issues, 6th edition. Black Point, NS: Fernwood, pp. 260–284.

[cars12491-bib-0044] Sheehy, E.A. & Ostridge, L. (2022) Making universities safe for women: sexual assault on campus. In: Antony, W. , Antony, J. & Samuelson, L. (Eds.) Power and resistance: critical thinking about Canadian social issues, 7th edition. Black Point, NS: Fernwood, pp. 318–348.

[cars12491-bib-0045] Smith, D. (2005) Institutional ethnography: a sociology for people. Lanham, MD: AltaMira.

[cars12491-bib-0046] Smith, D. (2006) Institutional ethnography as practice. Lanham: Rowman & Littlefield.

[cars12491-bib-0047] Smith, D.E. & Griffith, A.I. (2022) Simply institutional ethnography: creating a sociology for people. Toronto: University of Toronto Press.

[cars12491-bib-0048] Smith, G.W. (1988) Policing the gay community: an inquiry into textually mediated relations. International Journal of Sociology and Law, 16, 163–183.

[cars12491-bib-0049] Stermac, L. , Horowitz, S. & Bance, S. (2017) Sexual coercion on campus: the impact of victimization and disclosure on the educational experiences of Canadian women. In: Quinlan, E. Quinlan, A. , Fogel, C. & Taylor, G. (Eds.) Sexual violence at Canadian universities. Waterloo: Wilfred Laurier University Press, pp. 27–44.

[cars12491-bib-0050] Towl, G. (2016) Tackling sexual violence at UK universities: a case study. Contemporary Social Science, 1(4), 432–437.

[cars12491-bib-0051] Trusolino, M. (2017) It's not about one bad apple: the 2007 York University Vanier residence rapes. In: Quinlan, E. , Quinlan, A. , Fogel, C. & Taylor, G. (Eds.) Sexual violence at Canadian universities. Waterloo: Wilfred Laurier University Press, pp. 79–91.

[cars12491-bib-0052] Tummons, J. (2017) Institutional ethnography, theory, methodology, and research: some concerns and some comments. In: Reid, J. & Russell, L. (Eds.) *Perspectives on and from institutional ethnography* (*studies in qualitative methodology)* , vol. 15. Bingley: Emerald Publishing Limited, pp. 147–162. Available from: 10.1108/S1042-319220170000015003

[cars12491-bib-0053] Turner, S.M. (2006) Mapping institutions as work and texts. In Smith, D. (Ed.) Institutional ethnography as practice. New York: Rowan & Littlefield Publishers, pp. 139–161.

[cars12491-bib-0054] University and College Union . (2023) Precarious work in higher education . UCU. Available at: https://www.ucu.org.uk/media/14007/Precarious‐work‐in‐higher‐education‐update‐August‐2023/pdf/.

[cars12491-bib-0055] Whitley, L. (2020) How contingent faculty contracts contribute to sexual violence on campus . Gender Policy Report. Available at: https://genderpolicyreport.umn.edu/how‐contingent‐faculty‐contracts‐contribute‐to‐sexual‐violence‐on‐campus/. Accessed 1 May 2024.

[cars12491-bib-0058] Williams, P. (1991). The alchemy of race and rights: Diary of a law professor . Cambridge: Harvard University Press.

[cars12491-bib-0056] Yung, C.R. (2015) Concealing campus sexual assault: an empirical examination. Psychology, Public Policy, and Law, 1(1), 1–9.

