# Supplementary material for: Sexual violence, secrets, and work: Ruling relations of campus sexual violence policy
Source: Can Rev Sociol. 2025 Jan 7;62(1):34–54. doi: 10.1111/cars.12491 (PMC11830407; doi:10.1111/cars.12491)
Supplement: Supplementary file 1 — Supporting information [file CARS-62-34-s002.pptx]

## Slide 1
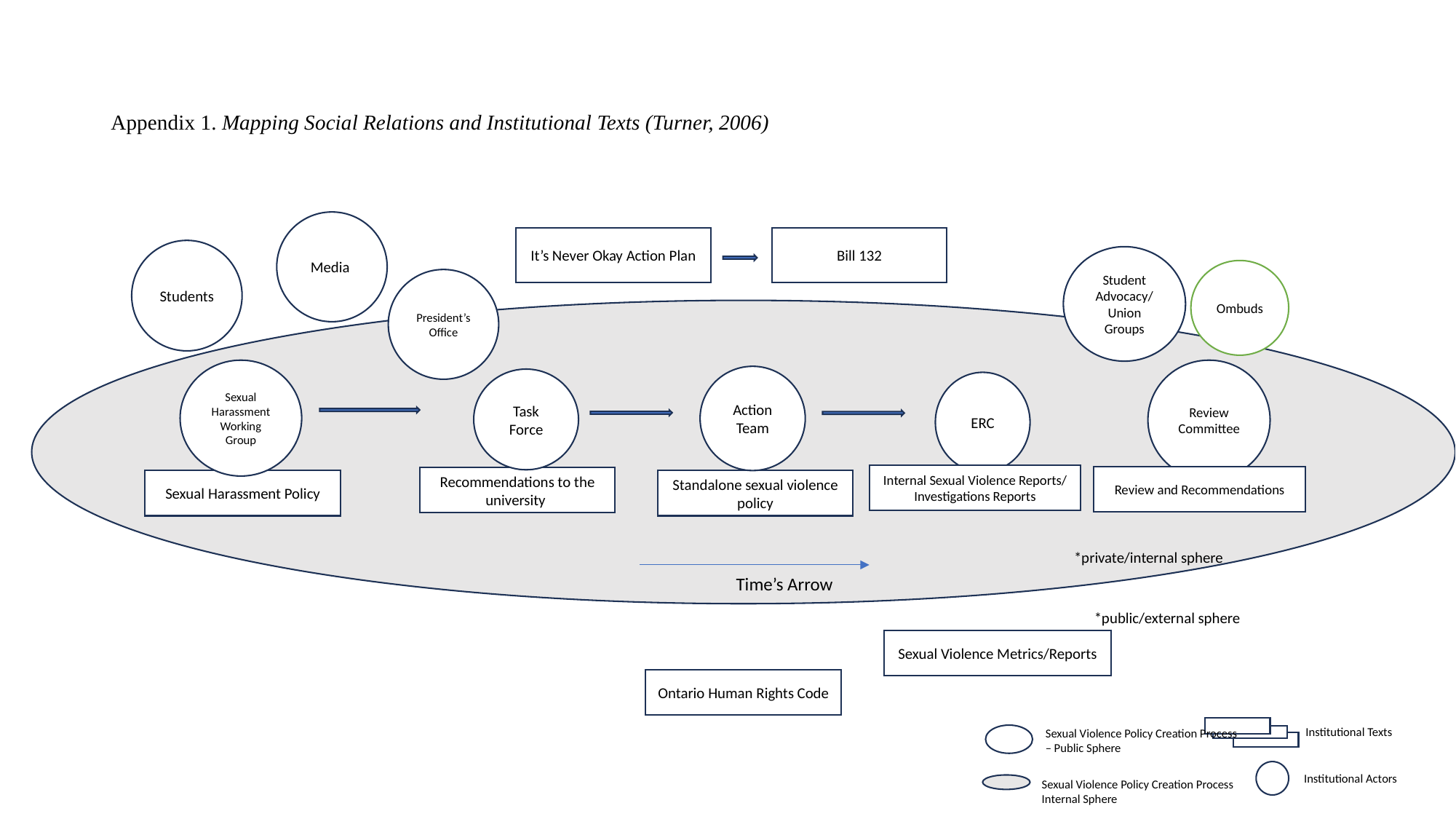

# Appendix 1. Mapping Social Relations and Institutional Texts (Turner, 2006)
Media
It’s Never Okay Action Plan
Bill 132
Students
Student Advocacy/
Union Groups
Ombuds
President’s Office
Review Committee
Sexual Harassment Working Group
Action Team
Task Force
ERC
Internal Sexual Violence Reports/ Investigations Reports
Review and Recommendations
Recommendations to the university
Sexual Harassment Policy
Standalone sexual violence policy
*private/internal sphere
Time’s Arrow
*public/external sphere
Sexual Violence Metrics/Reports
Ontario Human Rights Code
Institutional Texts
Sexual Violence Policy Creation Process – Public Sphere
Institutional Actors
Sexual Violence Policy Creation Process
Internal Sphere

## Slide 2
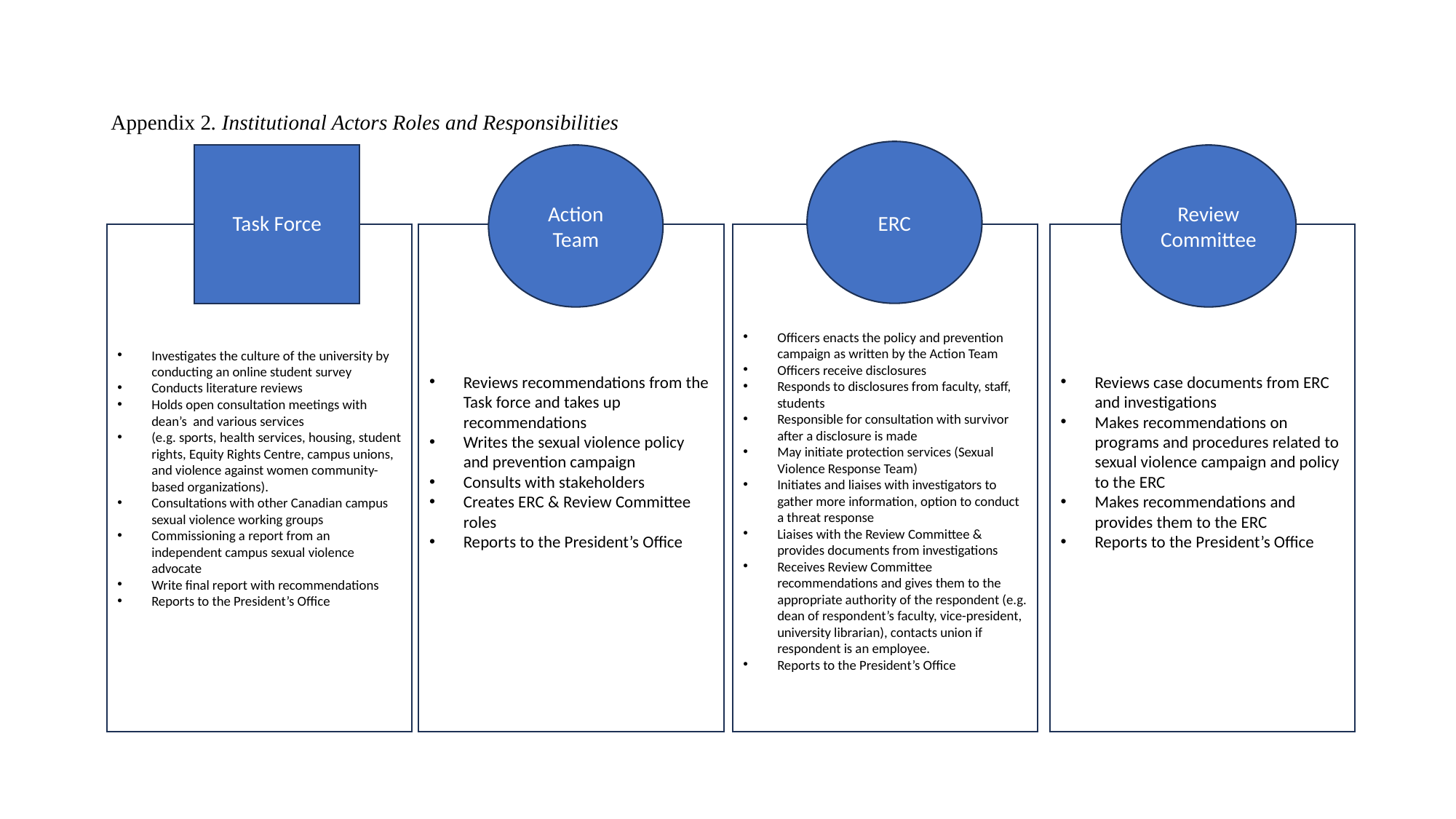

# Appendix 2. Institutional Actors Roles and Responsibilities
ERC
Task Force
Review Committee
Action Team
Investigates the culture of the university by conducting an online student survey
Conducts literature reviews
Holds open consultation meetings with dean’s and various services
(e.g. sports, health services, housing, student rights, Equity Rights Centre, campus unions, and violence against women community-based organizations).
Consultations with other Canadian campus sexual violence working groups
Commissioning a report from an independent campus sexual violence advocate
Write final report with recommendations
Reports to the President’s Office
Reviews recommendations from the Task force and takes up recommendations
Writes the sexual violence policy and prevention campaign
Consults with stakeholders
Creates ERC & Review Committee roles
Reports to the President’s Office
Officers enacts the policy and prevention campaign as written by the Action Team
Officers receive disclosures
Responds to disclosures from faculty, staff, students
Responsible for consultation with survivor after a disclosure is made
May initiate protection services (Sexual Violence Response Team)
Initiates and liaises with investigators to gather more information, option to conduct a threat response
Liaises with the Review Committee & provides documents from investigations
Receives Review Committee recommendations and gives them to the appropriate authority of the respondent (e.g. dean of respondent’s faculty, vice-president, university librarian), contacts union if respondent is an employee.
Reports to the President’s Office
Reviews case documents from ERC and investigations
Makes recommendations on programs and procedures related to sexual violence campaign and policy to the ERC
Makes recommendations and provides them to the ERC
Reports to the President’s Office
