# Supplementary material for: Sexual violence, secrets, and work: Ruling relations of campus sexual violence policy
Source: Can Rev Sociol. 2025 Jan 7;62(1):34–54. doi: 10.1111/cars.12491 (PMC11830407; doi:10.1111/cars.12491)
Supplement: Supplementary file 2 — Supporting information [file CARS-62-34-s001.docx]

Appendix 3*. Participant Demogrphics*

1. Samantha, a white lesbian cisgendered women, student union representative
2. Arathy, queer, cis-gendered woman of colour, Equal Rights Officer
3. Sophia, white, heterosexual, cisgendered female professor on the task force
4. Anna, queer cisgendered woman of colour, anti-racism activist and student union representative in the action group
5. Charlotte, a white, heterosexual cisgendered legal professor, in the action group
6. Caroline, a white heterosexual cis-gendered woman, paid social justice advocate.
7. Lydia, white, heterosexual cisgendered, female, trained lawyer, ombudswoman
8. Theodore, white, heterosexual cisgendered male, student rights advocate, lawyer
9. Peter, white, heterosexual, cisgendered male, paid employee, student rights advocate
10. Ava, a queer, cis-gendered woman of colour, front-line response worker at the women’s center.
11. Emma. white, heterosexual cisgendered woman, Equal Rights Officer
12. Daniel, white, queer cisgendered male student volunteering on the review committee
13. Anthony, white, heterosexual, cisgendered male, Senior Administrator
